# What motivates the choice to custom hire pest management spraying services?

**DOI:** 10.1002/ps.8494

**Published:** 2024-11-12

**Authors:** Braeden Van Deynze, Trey Malone

**Affiliations:** ^1^ Washington Department of Fish and Wildlife Washington United States; ^2^ Department of Agricultural Economics Purdue University Indiana United States

**Keywords:** custom hiring, timeliness costs, risk aversion, pest management

## Abstract

**BACKGROUND:**

This article presents a model of how farmers choose to custom hire for pest control. The decision‐making process is illustrated through a discrete choice experiment conducted via a pilot survey of soybean growers in Michigan, Illinois, and Indiana. Farmers responded to a hypothetical pest infestation by choosing between custom operators, spraying on their own, or leaving the field to its fate.

**RESULTS:**

Among farmers who choose to spray, the mean willingness to pay for marginal increases in timeliness (as defined as the chance of late spraying) ranges from 37 to 52 cents per acre. We also find that farmers more averse to risk are more sensitive to custom operator timeliness and that farmers with better‐developed social networks are less sensitive to the risk of delay.

**CONCLUSION:**

The results of this study can motivate future research into the drivers of on‐farm decision‐making, especially as it relates to custom hire behavior in pest control and other field operations. © 2024 The Author(s). *Pest Management Science* published by John Wiley & Sons Ltd on behalf of Society of Chemical Industry.

## Graphical abstract

A farmer's choice to custom‐hire pest management services is influenced by timeliness and risk aversion. Those more averse to risk value timely service highly, while strong social networks mitigate concerns about delays, making custom hiring more appealing.

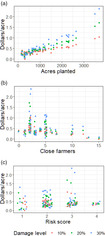

## 1 INTRODUCTION

What makes farmers choose to custom hire pest management spraying services while their neighbor chooses ownership? While the need for large and often expensive agricultural machinery is ubiquitous for fieldwork activities necessary to produce field crops, assigning property rights over such investments often differs from operation to operation. Some farmers choose to invest in and operate such machinery themselves. In contrast, others hire custom operators who own and operate their machinery to complete specific machinery‐intensive activities.

**Figure 1:**
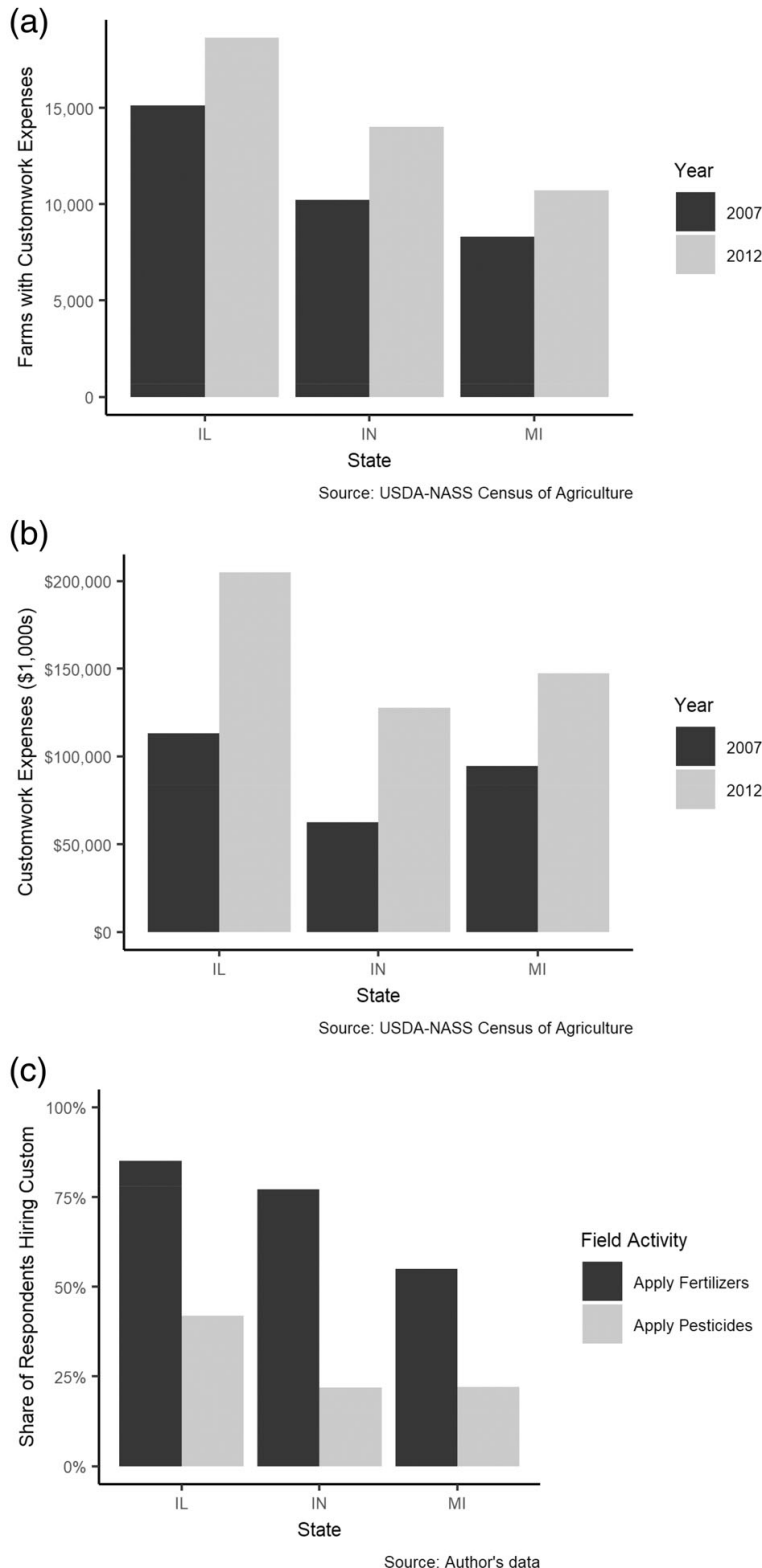
Custom hiring trends in three soybean‐growing states. Author's data collected in 2017 via a mail survey of 1478 soybean farmers across Illinois, Indiana, and Michigan.

This choice is especially important for pest management decisions, where custom hiring is used extensively by row crop farmers across the Midwest. Figure 1 presents trends for custom work. Between 2007 and 2012, the number of farms hiring custom for fieldwork in Illinois, Indiana, and Michigan increased by 23%, 37%, and 29%, respectively.^1^ Expenditures on custom work rose significantly during the same period, increasing by 75%, 104%, and 56% in Illinois, Indiana, and Michigan, respectively.^1^ The window for successfully applying insecticides is particularly important to the decision whether or not to outsource application responsibility, as insect pest populations can arrive unexpectedly and grow exponentially if left untreated.^2^ The effective completion of many field operations can also be unexpectedly limited by adverse weather.^3^ If pest control is not completed promptly, yield loss can be catastrophic, leading to potential yield losses of as much as 50%.^4^

Despite the importance of this decision‐making process, few studies have explored how producers choose to spray.^5^ This gap in the literature is surprising, as timeliness is vital to addressing the growing number of pesticide reduction initiatives.^6^, ^7^, ^8^ From the farmer's perspective, choosing custom contracting over vertical control can add another layer of uncertainty, as completing the task depends on another agent's actions under imperfect observability.

Farmers frequently outsource machinery‐intensive field operations to custom operators, though the use of custom operators varies widely by production task (e.g., planting, fertilizer application, pesticide application, harvesting). Among corn and soybean growers in Illinois, Indiana, and Michigan, custom operators are hired to apply fertilizers much more frequently than they are to apply pesticides.^9^ These tasks are distinguished by vulnerability to unexpected events, which cause lapses in work quality or timeliness and cause decreases in yields and farm profitability.^10^, ^11^

This article examines the role of uncertainty in driving transaction costs in pest control, which we expect can explain the relatively low rate of custom hiring (i.e., contracting) for this field operation. Beyond examining the role of uncertainty in increasing transaction costs, we also examine the role of social capital in mitigating such costs by providing information networks and reputational punishment mechanisms to distinguish between trustworthy and untrustworthy custom operators.^12^ After formulating hypotheses of the potential drivers of the custom hire decision, we present results from a discrete choice experiment conducted with 65 soybean growers from across Illinois, Indiana, and Michigan. Our primary goal here is to encourage further research into this decision task rather than to present our results as definitive willingness‐to‐pay estimates. As such, our empirical results should be used to inform future research into growers' contracting decisions, both for pest control and other on‐farm services.

## 2 MATERIALS AND METHODS

While little work has been done on the decision to spray, other farmer‐focused studies have examined drivers of choices to contract during other stages of the production cycle. Some studies focus on the choice to access different marketing channels and the characteristics of the contracts that govern them.^13^, ^14^, ^15^, ^16^ Others focus on controlling property rights and contracting characteristics for arable land.^17^, ^18^, ^19^ These studies model the choice to contract or contract use as a function of variables that drive or mitigate transaction costs. Similarly, a farmer's decision to custom hire parallels a traditional firm's choice between producing its own inputs or procuring them via contracting with another firm.^20^ Asset specificity exists within a specific time frame for pesticide application, as once an economically significant pest infestation is recognized, there is a critical period during which the infestation can be treated before risking significant yield loss. The value of these losses due to late treatment is called timeliness costs.^10^ However, the exact dates during the growing season when pests will approach economically damaging levels, or whether a pest infestation will occur at all, is impossible to know *a priori*.^4^

When custom hiring, farmers forfeit control over when and where the sprayer is used, which can increase the likelihood of delays in treatment, amplifying potential timeliness costs.^10^ Farmers who own and operate their own sprayer can more readily apply pesticides precisely when and where they are needed once uncertainty regarding a potential pest infestation is resolved. The custom operator may have other customers with pest infestations simultaneously occurring and must choose whose field to treat first. Random occurrences that would lead to delays even if the farmer chose to spray on their own, like unexpected weather, are amplified if they chose to custom hire as they further increase the likelihood of overburdening the custom operator. Because of the high degree of uncertainty, limited optimal treatment window, and large potential yield losses surrounding pest control, timeliness costs have the potential to be sizable.

We introduce uncertainty over whether pest control will be completed within the optimal window, linked with key producer concerns such as consistency and expected yield loss.^21^ The probability of on‐time pest control for custom operators is related to trust. Bhattacharya *et al*.^22^ proposed a formal definition of trust expressed verbally as ‘an expectancy of positive (or nonnegative) outcomes that one can receive based on the expected action of another party in an interaction characterized by uncertainty.’ In other words, people hold ‘conjectures’ about other people, defined as the probability from the person's perspective that the other person will complete specific actions.^22^ These conjectures matter for the cost of timeliness, which is composed of the probability of delay, the damage from delay, and the yield potential. We anticipate that when the damage from delay is larger, custom operators are less likely to be hired. Furthermore, the custom operator will be less likely to be hired when the probability of a custom operator being delayed in spraying is higher.

### 2.1 Risk aversion

Farmers have a wide spectrum of risk aversion, with some preferring non‐stochastic options to stochastic alternatives with equivalent expected profit.^23^, ^24^ Each custom operator can be considered a lottery, with payoffs and conjectures about the reliability of the custom operator serving as probabilities for each outcome. Not spraying and spraying on one's own are essentially degenerative lotteries. By including a risk attitude, we allow for a variety of possible behavioral theories, including the curvature of the utility function,^25^ loss aversion and probability weighting schemes,^26^ and models that do not rely on weighted averages of outcomes and allow uncertainty to directly affect utility.^27^ If some farmers are more averse to risk than others, this should be reflected in their custom hiring decisions, and more risk‐averse farmers will be less likely to select risky options. As such, we hypothesize that the more risk‐averse farmers are more sensitive to potential delays in spraying.

The decision to opt for custom spraying *versus* self‐spraying can also be viewed as a function of the relationship between the farmer, the operator, and the circumstances surrounding the choice. However, this decision ultimately hinges on whether the farmer decides to spray at all. Personal relationships and social networks can reduce transaction costs by easing the flow of information and establishing informal punishment systems for those who violate norms.^28^ For example, empirical research analyzing over 3000 cropland rental contracts, both formal and informal, from Nebraska and South Dakota finds evidence supporting reputational enforcement mechanisms in which farmers with more developed social networks are more likely to participate in informal (i.e., unwritten) contracts.^18^ For example, Bakker *et al*.^29^ found that a farmer's intention to reduce pesticide use is linked to whether other farmers in their network also choose to reduce pesticide use.

A similar social capital mechanism may exist in the context of custom pest control. Farmers who have larger peer networks can rely on other farmers for additional information about the custom operator's reliability and easily spread the news of late spraying by a specific operator. Therefore, the probability that a custom operator delays pesticide application may depend on the social network of the farmer contracting for the work. When farmers have more social capital, they can rely on these networks to punish custom operators who provide late service by damaging their reputation among potential customers. All else being equal, such punishment introduces additional costs to the operator for spraying late. Therefore, from the farmer's perspective, any given operator is less likely to provide late service, and farmers are likely to weight probabilities of delay downward. We hypothesize that farmers with a more developed social network are less sensitive to potential delays in spraying by custom operators.

### 2.2 Experimental design

Our empirical approach hinged on a choice model in which a farmer faces an acute pest infestation and chooses among three possible responses: (1) spraying with their own equipment, (2) hiring a custom operator to spray for them, or (3) not spraying at all. We then developed decision models to provide insight into the tradeoffs involved in custom hiring for pest control and the characteristics of farmers who might be more likely to custom hire.^1^

If an insecticide is applied late, the pest inflicts damage as a proportion of the field's yield potential. Farmers must choose to spray with their own sprayer, custom hire, or not to spray at all and accept the damages. Each alternative has an expected profit.^2^ The crucial difference between spraying options is the alternative‐specific treatment cost, typically due to chemicals, labor, equipment, and fees. For our purposes, insecticides and chemical costs are incurred under both alternatives and, therefore, ignored in the comparative analysis. When farmers choose to spray themselves, additional costs include labor, the wages (or equivalently, the opportunity cost of time) of the farmer applying the chemicals, and the cost of owning and operating the sprayer. Equipment costs include fuel, maintenance, depreciation, and any costs involved with procuring a sprayer, such as search and rental costs if one is not readily available. Because the farmer's window of time to react to the infestation is limited, these procurement costs can be prohibitively high if a farmer has not made prior arrangements via long‐term rental or ownership of a sprayer. If a farmer chooses to contract hire, the only additional cost is the amount paid to the operator in return for services.

We designed a discrete choice experiment (DCE) to illustrate our model of custom hiring for pest management. By asking participants to choose between experimentally selected scenarios, this approach allows us to gather choice data on important decisions even when they occur infrequently, allowing us to observe the full choice set. In this DCE, farmers were asked to imagine that a generic, unspecified insect pest infests their largest soybean field. The characteristics described for the hypothetical pest were like soybean aphid, though the species was not mentioned by name. Soybeans were selected as a model crop because soybean farmers were likely to have recent experiences with acute pest infestations during the spread of soybean aphids in the mid‐2000s. Farmers were presented with the option of hiring one of three custom operators to spray, spraying themselves with their own equipment, or not spraying at all. While we did not specifically ask how long respondents had owned their sprayers, future research could benefit from exploring how equipment ownership duration influences the decision to custom hire, particularly among those who reported custom hiring for field operations despite having the necessary equipment themselves.

Farmers were presented with chemical spraying costs (in dollars per acre) and expected soybean prices (in dollars per bushel). These attributes remained fixed for all farmers through all choice scenarios. Farmers were told that they would be responsible for chemical costs in all spraying options (as is typical in custom pesticide application contracts) and instructed to assume that all custom options were available even if those specific options were not present in their area. The soybean price and chemical cost values were selected such that spraying dominates not spraying for a risk‐neutral farmer in the scenario with the lowest yields and the highest damage. Therefore, this design focused on who sprays, though the option to not spray and allow damage to occur remained.

**Figure 2:**
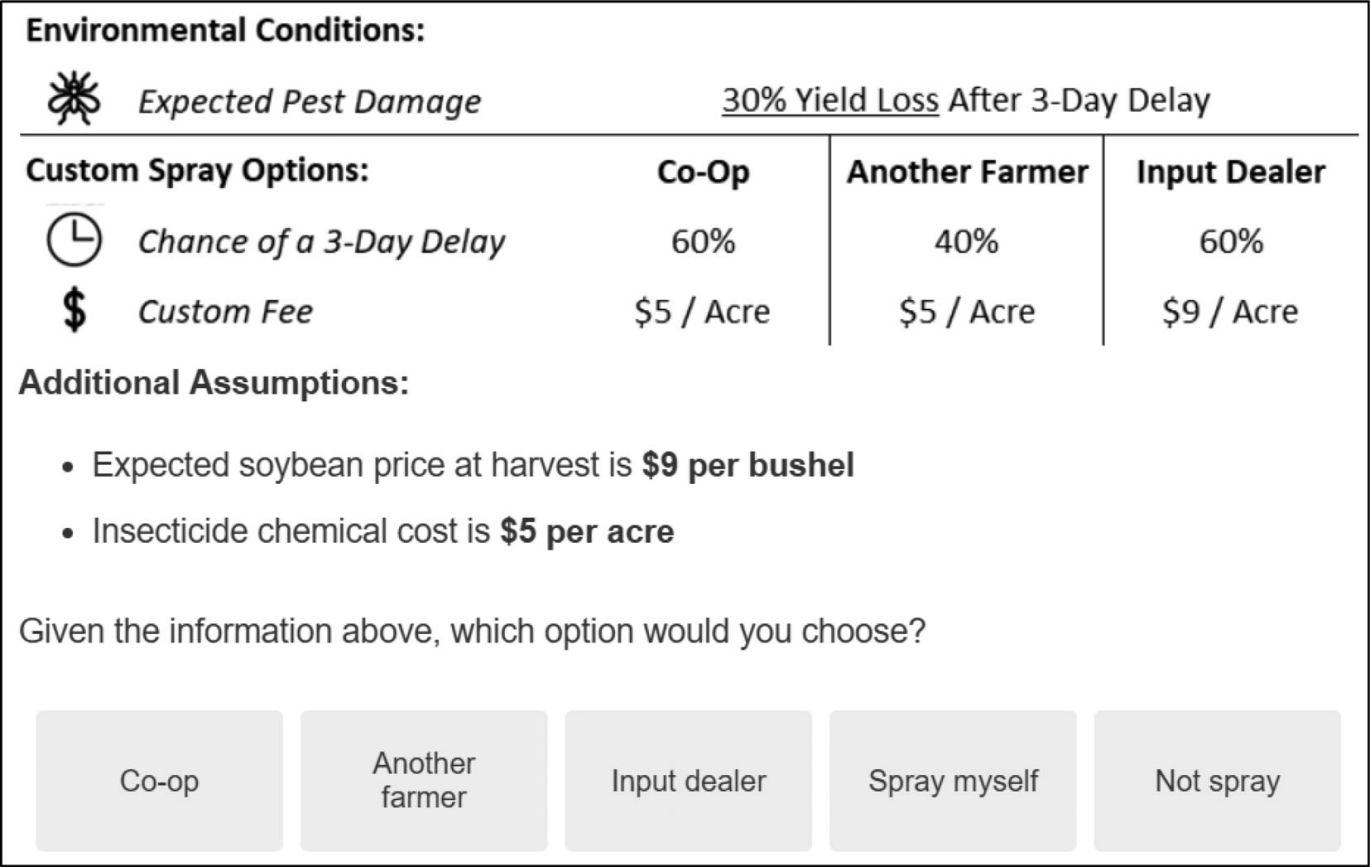
Example choice scenario.

Respondents each completed eight choice scenarios, which included a specific expected pest damage attribute that referred to the portion of yield lost to insect damage if spraying occurs after a three‐day window. Within each scenario, each custom operator option was presented as one of the following: an agricultural cooperative (or ‘co‐op’), an agricultural input dealer (or ‘dealer’), or another farmer. These three classes of custom operators represent the most common providers of custom pest control services. All three options were presented in each choice scenario, along with a ‘spray myself’ option, where the farmer would treat the field with their own equipment and labor, and a ‘do not spray’ option, where the farmer would leave the field to its fate and damage would be guaranteed. Each custom choice had an associated custom fee, presented as a dollar per acre fee paid to the operator, and a percentage chance of a three‐day delay, representing the probability that the pest damage occurs due to late spraying. Fee levels were based on the range of custom spraying rates reported in extension survey reports from Ohio, Michigan, Illinois, Iowa, and Indiana. Levels for each variable included in the choice experiment are presented in Table 1. An example of a choice scenario, as seen within the survey, is presented in Fig. 2.

**Table 1:** Levels and descriptions of choice experiment attributes

Attribute	Description	Value(s)
*Fixed*		
Chemical costs	Costs of insecticides used to spray, measured in dollars per acre	$5/ac
Soybean price	Price of soybean at harvest, measured in dollars per bushel	$9/bu
*Scenario*		
Pest damage	Damage the insect pest would induce if spraying is delayed three days, measured in portion of yield potential (d)	10%, 20%, 30% (3)
*Choice*		
Custom fee	Fee paid to operator for services, measured in dollars per acre (fee)	$5/ac, $9/ac, $13/ac (3)
Chance of delay	Probability spraying occurs three days late and pest damage occurs, measured as a percentage (p)	20%, 40%, 60% (3)
Operator identity	Identity of the custom operator	Co‐op, input dealer, another farmer (3)

A 24‐row fractional factorial experimental design was generated using the software package Ngene and split into three blocks of eight scenarios each. After consulting subject‐matter experts during the design stage, eight choice scenarios were determined to be the maximum feasible number of scenarios per farmer. Budget considerations preempted pilot data collection, which is necessary for using priors in generating designs targeting efficiency criteria. Because of these constraints, the design was generated by randomly selecting 24 rows from the full factorial design. Respondents were presented with an additional ‘cheap talk’ page in our study, encouraging them to take their time and respond as if their choices would have real impacts on their farms to mitigate hypothetical bias.^31^, ^32^

The survey was piloted with 20 professionals in the agricultural community unassociated with the study, including employees of the Michigan Department of Agriculture and Rural Development, Michigan State University Extension, and the Michigan Soybean Promotion Committee, as well as active farmers. Comments from phone and email interviews with reviewers were incorporated into the survey design to improve clarity and ensure the choice experiment represented a feasible scenario.

To test our experimental design and collect illustrative results a proof‐of‐concept for the hypotheses presented above, we conducted a small pilot with farmers with 100 or more acres of soybeans planted in 2017 in Michigan, Indiana, and Illinois, focusing on Michigan farmers. We contacted potential respondents via email and postal mail, randomly assigning a block to each respondent in a balanced manner. In total we received a response from 65 farmers across the three target states. Two blocks were completed 22 times, while the third was completed 21 times. Farmers were also asked questions about their past spraying and custom hiring activities, their capacity to spray with on‐farm equipment, and the characteristics of their farms. Farmers were also asked about their general attitudes towards trust and risk, and the number of other farmers with whom they are comfortable discussing important business matters. The full survey instrument is provided in Appendix S1. Note that due to the small number of observations, the empirical results that follow should be interpreted as illustrative of the use of DCE designs for testing the presented hypotheses, rather than representative measurements of preferences among the target population.

### 2.3 Econometric analysis

We estimate a series of conditional logit models, which measure farmer preferences for the characteristics of our decision alternatives as they relate to the choice to spray. Recent applications of these models have considered cases of uncertainty over whether an alternative possesses one or more characteristics, often by including the probability that an alternative possesses a characteristic as a characteristic itself. Applications include measuring preferences for environmental quality where the outcome of a project is uncertain^33^, ^34^ and preferences for the travel time reliability of transportation options.^35^, ^36^ In these studies, the possible levels of the characteristic and the probability of the level occurring influence how each alternative affects utility. The two frequently interact to capture changes in the expected value of the uncertain characteristic.

In our setting, the uncertain characteristic is whether the custom applicator arrives on time and prevents the pest from damaging the crop. This uncertainty is present only for the custom options. When a farmer chooses to spray on their own, the probability of delay is assumed to be zero. When they choose not to spray, the probability is assumed to be 1 (i.e., the damage is guaranteed), consistent with the framing in the choice experiment and the conceptual model.

In our setting, potential pest damage to the farmers' crop is measured in bushels per acre and computed as the percentage of yield loss unique to the choice occasion multiplied by their expected pest‐free soybean yield.

By including an interaction term with these expected damages, we partly modeled timeliness costs, assuming that farmers are risk‐neutral and that the utility effects of damage and probability of delay are inseparable. We also estimated an alternative model that allows the probability of delay to affect utility through the effect on expected damage and a direct effect on utility itself.

Including a separate term for timeliness, apart from the interaction, allowed us to capture any residual preference for timeliness that is not fully explained by the interaction term. If farmers are risk‐neutral and weigh damage exactly according to the probability of its occurrence, then the parameter would be estimated at 0. A negative estimate would suggest that farmers are risk‐averse or have a distaste for uncertainty beyond its effects on increasing expected damage, indicating risk aversion or other behavioral distortions from the simple risk‐neutral weighting of outcomes. This model also allows for an ‘uncertainty effect’ in which farmers discount risky alternatives because of the existence of risk itself rather than because of the effects of risk on the expected utility of the alternative.^27^

These specifications were also considered against a linear model specification in the choice attributes, which assumes that all interaction effects are zero and that farmers do not condition yield outcomes by the probability of their occurrence.^34^ While this is an extreme assumption, the model was retained as a test that such probability conditioning occurs. We used multiple criteria to select a preferred model, including likelihood ratio tests, Akaike's information criteria (AIC), and Bayesian information criteria (BIC).

### 2.4 Farmer preference heterogeneity

On completing our base model selection process, we introduced farmer characteristics to examine how farmer sensitivity to delay varies and illustrate hypotheses related to farmer characteristics. These variables can interact with the characteristics of the alternatives so that the resulting coefficients can vary by the characteristics of the respondent. We divided characteristics into two classes: characteristics that affect utility under custom options and characteristics that affect utility when the farmer chooses to spray on their own.

For custom option characteristics, we included ‘number of close farmers’ as a proxy of social capital, which measures the number of other farmers, excluding those associated with the respondent's operation, with whom the farmer feels ‘close enough to discuss important business problems.’ To test whether more risk‐averse farmers are wary of custom hiring, we included ‘risk score,’ the farmer's self‐rating on a four‐point scale where 1 is defined as ‘fully prepared to take risks’ and 4 is defined as ‘unwilling to take risks.’^3^ To examine how farms of different sizes respond differently to uncertainty, we included ‘acres planted,’ which measures the total acres the farmer planted in 2018 of soybeans or any other crop. We interacted each selected farmer characteristic for a three‐way interaction to examine the effects of each characteristic on farmer preferences for timeliness in custom hiring.

For characteristics relevant when the farmer sprays on their own, we included variables that proxy for costs incurred when that alternative is selected. ‘Farm income share,’ measured as a proportion of total income, proxies for the opportunity cost of labor, with a higher proportion of income from off‐farm sources assumed to indicate a higher opportunity cost of on‐farm labor. This variable tests whether higher labor costs lead to more frequent custom hiring. We also interacted this variable with the alternative specific constant for ‘spray myself’ to capture variation in preferences for spraying using one's own labor.

To aid in interpreting the coefficients for these interactions, we centered each non‐binary characteristic at 0 by subtracting the sample mean. Therefore, the resulting coefficients can be interpreted as piecewise utility changes resulting from a unit change from the mean.

### 2.5 Choices by sprayer ownership

To illustrate that farmers with their own equipment are more likely to spray on their own, we compared the frequency that ‘spray myself’ was selected among alternatives between respondents who report owning or leasing a self‐propelled sprayer, those who own a tractor‐pulled sprayer and those who do not own a sprayer at all. Self‐propelled sprayers are specialized equipment that can apply chemicals over larger areas more quickly and at lower equipment and labor costs than tractor‐pulled sprayers. While this method cannot distinguish between equipment and labor cost savings resulting from sprayer ownership, it provides an equipment gradient over which we can examine differences in the likelihood that custom hiring is selected.

## 3 RESULTS

In total, 65 farmers completed surveys for 519 choice scenarios (one choice scenario was incomplete). Most responding farmers reported possessing the equipment and certification to perform the tasks independently. Seventy‐five percent of respondents were certified to spray restricted‐use pesticides, and 69% owned or leased their own sprayer (Table 2). Insecticides were used infrequently among the respondents. The median respondent sprayed insecticides twice in the past 10 seasons and twice on soybeans specifically (Table 3). When spraying does occur, 32% of respondents typically hired custom when spraying was needed.

**Table 2:** Summary of categorical survey variables

Variable	Description	Category	*N* ^a^	% of sample^b^
Who sprays	Who sprays insecticides in a typical year	Custom applicator	21	32%
		Employee (family excluded)	2	3%
		Family member	5	8%
		Primary operator (myself)	34	52%
		Other	3	5%
Certification	Whether anyone who works on the farm is certified to spray restricted use insecticides	No	16	25%
		Yes	49	75%
Sprayer ownership	Whether the farm owns a sprayer	No	20	31%
		Yes	45	69%
Farm revenue	Gross farm income in 2017	Less than $150 000	8	12%
		$150 000–$349 999	25	39%
		$350 000–$999 999	16	25%
		$1 000 000–$4,999 999	8	12%
		More than $5 000 000	1	2%
		No Response	7	11%
Gender	Farmer's gender	Female	5	8%
		Male	56	86%
		No Response	4	6%
Education	Farmer's level of education	High school graduate	15	23%
		Some college	12	19%
		Two‐year degree	14	22%
		Four‐year degree	18	28%
		Professional degree	5	8%
		No response	1	2%
Household income	Farmer's household income in 2017	Less than $20 000	4	6%
		$20 000–$39 999	2	3%
		$40 000–$59 999	7	11%
		$60 000–$79 999	6	9%
		$80 000–$99 999	10	15%
		More than $100 000	26	40%
		No response	10	15%
State	Farmer's state of residence	IL	10	15%
		IN	22	34%
		MI	33	51%

^a^ Number of responses to each item, accounting for item non‐response. Total number of responses is 65.

^b^ Percentages may not add to 100% due to rounding.

**Table 3:** Summary of numeric survey variables

Variable	Description	Min.	25th‐Perc.	Med.	75th‐Perc.	Max.	Mean	Std. Dev.	*N* ^a^
Past custom	Years in the last 10 when custom work was used	0	0.5	5	10	10	5.5	4.3	63
Past custom, spray	Years in the last 10 when custom spraying was used	0	0	1	3.5	10	2.6	3.3	63
Past spray	Years in the last 10 when any insecticides were used	0	1	2	5	10	3.4	3.1	63
Past spray, soybeans	Years in the last 10 when insecticides were used on soybeans	0	1	2	3	10	2.7	2.6	59
Age	Age in years	27	54	59	66	80	58		
Close farmers	Not including those who work on your operation, about how many other farmers would you say you feel close enough to discuss important business problems with?	0	2	3	5	15	4.2	3.0	61
Risk score	Are you generally a person who is fully prepared to take risks or do you try to avoid taking risks? (fully prepared to take risks = 1; unwilling to take risks = 4)	1	2	2	3	4	2.3	0.8	64
Expected yield	Expected yield, in bushels per acre, of largest soybean field	35	53	57	65	81	58	9.3	64
Farming income	Percent of farmer's household income from agriculture	0	25	52	95	100	58	34.5	62
Planted acres	Total planted acres in 2017	150	409	697	1251	3700	997	804	64

^a^ Number of responses to each item, accounting for item non‐response. Total number of responses is 65.

Survey respondents routinely hired custom operators. The median respondent hired a custom operator for any field operation in five of the past 10 seasons and specifically for spraying pesticides in one of those seasons (Table 3). Seventy‐four percent of respondents reported custom hiring for at least one field operation in at least one of the past 10 seasons. Most respondents consulted with a small circle of other farmers on important issues, though some had larger networks. The median respondents were close enough to three other farmers to discuss important business issues, and a quarter were close enough to five or more other farmers to have such discussions (Table 3). Our sample, on average, expressed a slight preference for risk‐taking behavior, with a mean risk score of 2.3 (Table 3).^4^

Table 4 reports the unconditional rates for each alternative in the DCE. Respondents chose to spray with their own equipment most frequently (45.5% of choice occasions). Among custom options, input dealers were selected most frequently (24.5%), followed by co‐ops (15.2%), and other farmers (11.4%). Not spraying was selected rarely (3.5%).

**Table 4:** Response shares to the choice experiment

Alternative	*N^a^*	% of responses
Co‐op	79	15.2%
Input dealer	127	24.5%
Farmer	59	11.4%
Myself	236	45.5%
None	18	3.5%

^a^ Indicates statistical significance at the *α* = 5% level.

Number of respondents = 64 respondents.

Total number of choice occasions = 511.

Econometric results are presented in Table 5. By comparing model fit statistics, we can conclude that including a parameter for the probability of delay improves model fit over the base model. All coefficients for the preferred model are statistically significant at the *α* = 5% level or better. Consistent with economic theory, the model indicates that farmers custom hire less when that option is more expensive. The coefficient for expected damage is also negative, indicating that farmers are less likely to choose an option when the option's expected timeliness costs increase. The model also indicates that farmers have a distaste for the increased probability of delay separate from the effects on their potential yields. The alternative specific constants are all positive, indicating a residual preference for all spraying options over not spraying at all.

**Table 5:** Results of conditional logit models

	Model 1		Model 2	
Variable	Coefficient	SE	Coefficient	SE
Fee ($/acre)	−0.10*	0.02	−0.11*	0.02
Delay probability			−2.60*	0.47
expected damage, $p_{a}\mathrm{dY}$	−0.19*	0.03	−0.09*	0.02
ASC – farmer	0.08*	0.35	2.30*	0.37
ASC – co‐op	1.10*	0.33	2.80*	0.38
ASC – dealer	1.50*	0.34	3.10*	0.37
ASC – self	0.52	0.37	1.50*	0.32
Log likelihood	−661.5		−651.6	
AIC	1335.1		1317.1	
BIC	1360.5		1346.8	

*Note*: * indicates statistical significane at *α* = 5% level.

### 3.1 Results with farmer characteristics

Results with farmer characteristics are presented in Table 6. The core results for coefficients estimated without farmer characteristics remain consistent with the prior models, including the rankings of the alternative specific constants. The coefficient for ‘close farmers,’ which represents the number of other farmers a respondent feels close enough to discuss important business problems with, is particularly revealing. When this variable is interacted with the probability of delay, the positive and statistically significant coefficient suggests that farmers who have more extensive social networks are less sensitive to potential delays by a custom operator. This could occur within a social network because farmers with stronger ties to other farmers may have access to more timely and reliable information about the performance and reliability of custom operators. They can also leverage social pressure or reputational consequences, which might reduce their concern over potential delays. On the other hand, the negative coefficient for ‘close farmers’ interacted with expected damage implies that these farmers, while confident in their network's ability to mitigate delays, are more acutely aware of the risks of actual damage should those delays occur. This dual effect highlights the subtle role that social networks play in decision‐making: they provide a buffer against uncertainty but also heighten awareness of potential losses when that uncertainty materializes.

**Table 6:** Results of the preferred conditional logit model with interactions for farmer characteristics

Variable	Coefficient	SE
Fee, fee ($/acre)	−0.12*	0.025
Delay probability, $I_{a}^{\text{custom}}p_{a}$	−3.6***	0.74
Close farmers (count)	0.39***	0.15
Risk score (1–4, 4 is risk averse)	−0.016	0.48
Acres planted	−0.00021	0.00091
Expected damage, $p_{a}\mathrm{dY}$	−0.15***	0.053
Close farmers	−0.024**	0.011
Risk score	−0.073**	0.034
Acres planted	−0.00031***	0.000072
ASC – farmer	3.9***	0.69
ASC – co‐op	4.4***	0.70
ASC – dealer	4.8***	0.70
ASC – self	2.6***	0.66
Farm income share (proportion)	0.0066*	0.0036
Log likelihood	−527.9	
AIC	1083.7	
BIC	1141.9	
Choice occasions	471	
Respondents	59	

***, **, and * indicate statistical significance at the 1%, 5%, and 10% levels.

The coefficients for the remaining two characteristics interacted with timeliness cost variables, ‘risk score’ and ‘acres planted,’ are statistically insignificant when interacted with the probability of delay but negative and statistically significant when interacted with expected damage. The coefficient for the variable ‘farm income share’ interacted with the alternative specific constant for spraying on one's own is statistically significant and positive, though only at the 10% level, indicating that farmers who receive a larger share of their household income from agriculture are more likely to choose to spray on their own.

### 3.2 Choices by sprayer ownership

Among the 20 respondents who do not own or lease a sprayer, none chose to spray on their own on any choice occasion. Among the 24 respondents who own or lease self‐propelled sprayers and the 21 who own or lease a tractor‐pulled sprayer, spraying on one's own was selected in 76% and 54% of choice occasions. Unsurprisingly, these results suggest that respondents who own more specialized spraying equipment are less likely to custom hire for pest control.

### 3.3 Willingness‐to‐pay for timely spraying

We now consider farmers' willingness‐to‐pay (WTP) for reductions in a custom operator's probability of delay, conditional on choosing to spray. Because all characteristics that interacted with the probability of delay were centered at 0 before estimation and, therefore, had means of 0, their effects are omitted from this calculation. We divide by 100 to measure WTP, measured in dollars per percentage change in probability of delay per acre rather than dollars per probability unit. We found that, on average, farmers in our sample were willing to pay $0.37 per acre for a 1% reduction in the probability of delay when the potential damage rate was 10% of yield, $0.45 per acre when the potential damage rate was 20%, and $0.52 per acre when the potential damage was 30%. This WTP for a marginal change in the probability of delay represents 2.7%, 3.2%, and 3.7% of median custom pest control costs per acre as presented in the choice experiment ($9 per acre in custom fees and $5 per acre in chemical costs).

**Figure 3:**
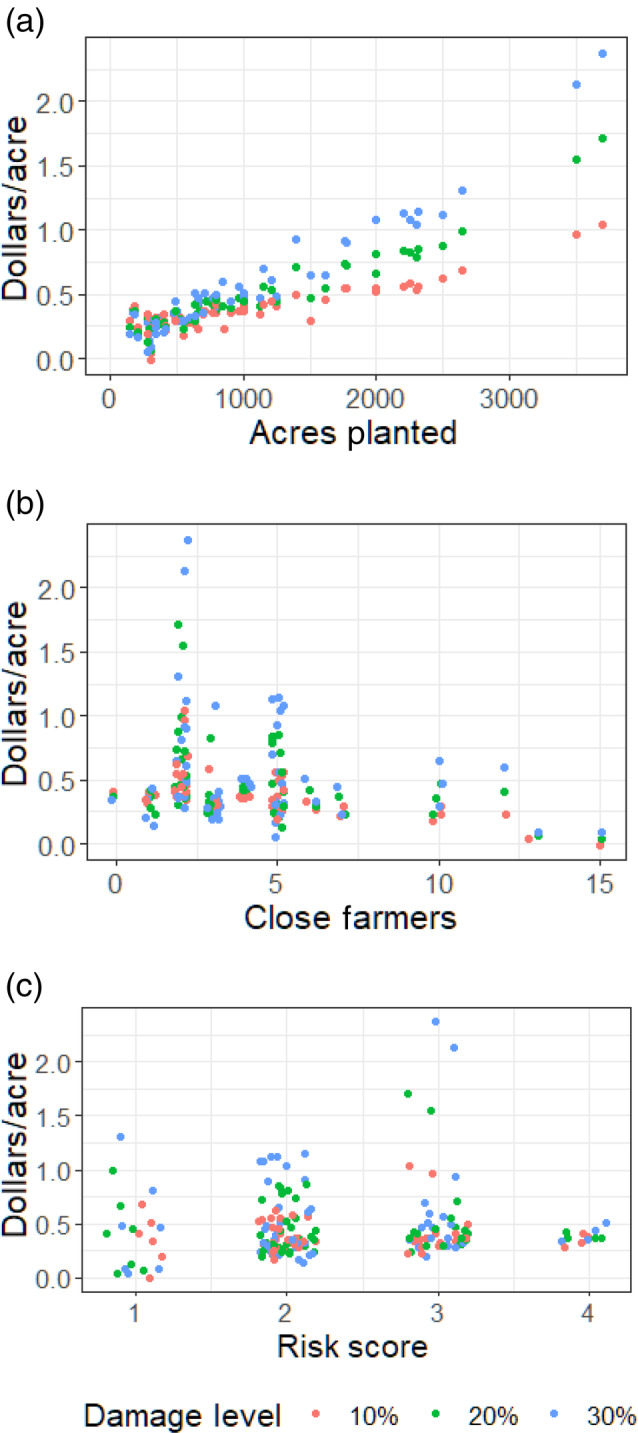
Willingness‐to‐pay (WTP) for reductions in the probability of delay. WTP is calculated for each respondent at each damage level and presented by (A) acres planted, (B) close farmers, and (C) risk score. Random displacement on the horizontal axis has been added to distinguish between overlapping points.

To show how farmer characteristics affect WTP to avoid delay, we illustrate WTP for each respondent in the sample according to farm size (Fig. 3(A)), the number of close farmers (Fig. 3(B)), and risk score (Fig. 3(C)). Respondent‐level WTP was calculated by adding the statistically significant interaction coefficients, multiplied by associated characteristics and potential damage levels, to the numerator of eqn (4).^38^ The potential damage rate was set at each of the three levels in the DCE to demonstrate the impact of increasing damage on WTP and avoid delay.

## 4 DISCUSSION

As predicted, higher custom fees reduce custom hire use among the pilot sample. However, we found only weak evidence that sampled farmers with higher opportunity labor costs, as measured by the proportion of household income derived from agriculture, are more likely to custom hire. In other words, if a farmer has the equipment, they will probably use it. Farmers with more specialized equipment (i.e., self‐propelled sprayers) are less likely to custom hire, implying that sprayer ownership decreases custom use. These results support the implication that farmers are more likely to custom hire when performing a task themselves is more expensive. Future research might explore whether labor or equipment costs are more important in driving this outcome, allowing for better predictions of how changes in labor and equipment markets might affect custom hiring demand. Our results also illustrate how farmers are less likely to custom hire when the expected damage is higher. Note that both increases in the probability of delay and the increase in the absolute level of damage can drive this effect.

Our results also support the notion that farmers do not respond to potential delays in custom hiring in a risk‐neutral way. The interaction coefficients from the base model suggest that risk‐averse farmers are more sensitive to the probability of delay, but only through its effect on expected damage. Future research should directly address alternative models of risk preferences when assessing drivers of custom hiring behavior among growers.

We address our social connectedness hypothesis by examining the coefficients for interaction terms, including the ‘close farmers’ variable. This aligns with a growing literature on disentangling perceptions from preferences in empirical decision models.^39^, ^40^ Our results indicate that farmers with broader social networks are less sensitive to increases in the probability of delay, in support of our hypothesis. While the coefficient for the interaction of ‘close farmers’ with expected damage is negative, the coefficient for the interaction with delay probability is large enough to counteract this negative effect. We interpret the coefficient for ‘close farmers’ interacted with delay probability as a mitigating effect of social capital. At the median potential yield damage (i.e., median pest‐free yield expectation of 57 bushels per acre multiplied by the median pest damage rate of 20%), the mitigating effect of social capital exceeds the negative impact on the marginal utility from expected damage. This comparison suggests that social capital ultimately mitigates the marginal disutility from the probability of delay for most farmers. In Fig. 3(B), there is a clear downward correlation between respondent WTP for reductions in the probability of delay and the number of farmers the respondent is close with.

The notion that farmers with closer relationships with other farmers are more likely to custom hire aligns with past findings that US farmers rely on informal social mechanisms to facilitate transactions that may otherwise not occur.^10^, ^12^, ^18^ While previous findings regarding the role of social capital in facilitating agricultural contracting focus on land, our results provide some evidence that such mechanisms persist in non‐land contexts. Future research might measure the relationships and social networks of custom operators and the farmers who hire them to explore further how social capital might support custom hiring markets. Relatedly, the size of the operation, farm income, and ownership are likely to be highly correlated. Because of the limited sample size, we cannot confidently parse these differences. That said, future research would benefit from comparing the WTP values of a sprayer ownership budget at the time of the survey. This would allow future studies to consider ownership breakeven costs as they relates to timeliness.

We also included ‘acres planted’ as a possible farmer characteristic influencing sensitivity to timeliness costs, finding that farmers who farm more land are more sensitive to expected damage. However, ‘acres planted’ did not influence sensitivity to delay probability separate from its impact on sensitivity to expected damage. There are multiple conflicting theoretical expectations regarding the effect of farm size on sensitivity to uncertainty when custom hiring. Larger farms represent larger revenue for a custom operator, often paid by the acre. If a custom operator sprays late, a farmer can withhold future business, and the custom operator would lose out on more revenue if that farm is large. Therefore, we might expect larger farms to underweight probabilities of delay similarly to those with more social capital, suggesting a positive expectation for this coefficient. By contrast, a larger farm requires more time to complete field operations. This finding might lead to increased sensitivity to the probability of delay because custom operators would struggle more to catch up if delays occur. Our null finding does not rule out either possibility. If both mechanisms mutually exist, their effects could counteract each other. Future research might closely examine the connection between farm size, transaction costs, and custom hiring decisions.

The alternative‐specific constants indicate a distinct ranking among alternative providers of custom services. The alternative‐specific constant is largest for input dealers, co‐op providers, and other farmers. All three custom alternatives have larger constants than the constant for the alternative in which farmers spray on their own, indicating that farmers would prefer a custom operator to spray if there were no custom fees and timely service was guaranteed. Note that in some cases in the study region co‐ops will buy and re‐sell chemicals from input dealers and input dealers themselves may not offer custom spraying services directly. Therefore, some respondents may have conflated co‐op and input dealer choices, which may be one possible explanation for the nearly identical alternative‐specific constant estimates for these two choices across model specifications.

Input dealers and co‐ops often offer additional services beyond custom spraying. These include agricultural inputs like fertilizers, seed, and pesticides and other custom services such as applying fertilizers or harvest. These additional services may explain the respondents' preference for custom spraying from input dealers and co‐ops over similar services from other farmers. When hiring custom spraying services, customers of input dealers and co‐ops can purchase other goods and services from these custom operators (or *vice versa*), reducing search costs. Another possible explanation for the ordering of preferences between custom service providers is that other farmers have their own fields, which may require treatment during the same critical window as their customers. Custom operators who manage their own farms have a clear incentive to prioritize their own fields over their customers, which may explain the preference among respondents for non‐farmer providers of custom spraying.

## 5 CONCLUSION

This article explores the drivers of custom pest control hiring decisions. The results from a pilot survey illustrate the importance of timeliness in these decisions. Using a discrete choice model of custom hiring in a pest control setting, we demonstrate how uncertainty over the reliability of custom operators can create timeliness costs, a specific type of transaction cost especially relevant in agricultural contexts.^10^ While previous studies have identified timeliness costs in custom hiring through case study methods, we provide preliminary empirical evidence that timeliness costs can drive farmers away from custom hiring and towards equipment ownership. Further, we illustrate that risk‐averse farmers might be more sensitive to these costs. Finally, we illustrate that farmers who are more integrated into the agricultural community (i.e., who have more close friends who farm) are less sensitive to timeliness costs.

Understanding which farmers opt to custom hire and which custom operators they choose when multiple providers are available can assist in identifying regions where demand for custom services may be high. Pest pressure dynamics, weather patterns, pesticide spraying regulations, and road infrastructure are all regional factors that can affect the ability of firms to provide timely services. For regions threatened by pests that affect nearby fields concurrently, many farmers will likely need to apply insecticides simultaneously. Farmers will likely find custom pest control unattractive in such a setting, as custom operators will be harder‐pressed to provide timely services to many farmers concurrently. Areas with highly variable weather are more likely to have unexpected delays in spraying for a given field, which might create backups in a custom operator's schedule. Problems created by weather will be exacerbated in states with stricter restrictions on weather conditions suitable for pesticide applications or regions where road conditions make moving between fields more difficult. Furthermore, a farmer's value of the input is likely to change over time, as the input only has value when needed.

Our empirical application is intended to illustrate mechanisms described in the conceptual model. Given the limited sample size, the results are not to be interpreted as representative measures of the preferences and behavior of soybean growers in Michigan, Indiana, and Illinois. A larger, more extensive survey effort would be needed to estimate the preference parameters of the larger soybean‐growing population. While this study focuses on pest control, it is worth noting that the machinery used for pest control often overlaps with other farm operations, such as fertilizing, where similar risk preferences and timeliness concerns may apply. This overlap could further compound a farmer's desire to own the equipment, as the same risk management considerations would influence multiple production decisions. Approaches building on this first attempt to characterize the drivers of custom hiring can be applied to scenarios where farmers custom hire for services other than pest control, such as harvest or fertilizer application. These field operations are subject to other forms of ecological uncertainty, which may induce timeliness costs in unique ways. Future studies may want to examine the role of crop insurance in the spray decision, as an uninsured field would likely need more timely application than the uninsured field, especially if losses were catastrophic. Similarly, growth in cover crop and carbon sequestration programs will likely alter the long‐term decision process. The decision to transition from hiring custom operators to self‐spraying is likely a gradual, multi‐year process influenced by repeated negative experiences rather than an immediate response to a single adverse outcome. Dynamic modeling would be useful in future studies as increased input use might lead to higher WTP for timeliness, as the investment would likely alter a producer's sunk costs. Farms make decisions regarding custom hiring every year for various field operations, providing a rich context to test transaction cost theories and examine what conditions lead to various distributions of property rights between farms and their operators. Such studies would provide additional insight into how farmers view timeliness and other transaction costs in the context of different field operations.

## Supporting information


**Appendix S1.** Online survey instrument

## Data Availability

The data that support the findings of this study are available from the corresponding author upon reasonable request.

## Footnotes

^1^
We assumed all production costs unrelated to pest control are unaffected by the farmer's pest control decisions and are therefore omitted from the analysis.

^2^
The remainder of the analysis focuses on differences between self‐ and custom spraying. Producers will not spray at all if spraying costs reach a high enough economic damage threshold.^30^

^3^
Similar versions of this question have been shown to have strong predictive validity for risky behavior, and responses are simpler to collect in a survey setting than alternative measures of risk attitudes.^37^

^4^
While we do not claim that our sample is representative of all farmers, we compared the demographic statistics of our sample relative to equivalent measures reported by the USDA for the sampled population (Appendix B).
