# Supplementary material for: What motivates the choice to custom hire pest management spraying services?
Source: Pest Manag Sci. 2024 Nov 12;81(2):912–22. doi: 10.1002/ps.8494 (PMC11716371; doi:10.1002/ps.8494)
Supplement: Supplementary file 1 — Appendix S1. Online survey instrument [file PS-81-912-s001.docx]

**Appendix A: Online Survey Instrument**

The following pages contain images of the survey instrument. The survey was administered on via respondents’ web browsers and contained conditional logic leading to multiple versions. Notes have been added to explain where reactive elements exist and to clarify structure. Choice experiment blocks were removed for brevity. Unless otherwise noted, each panel represents a separate page of the survey instrument.


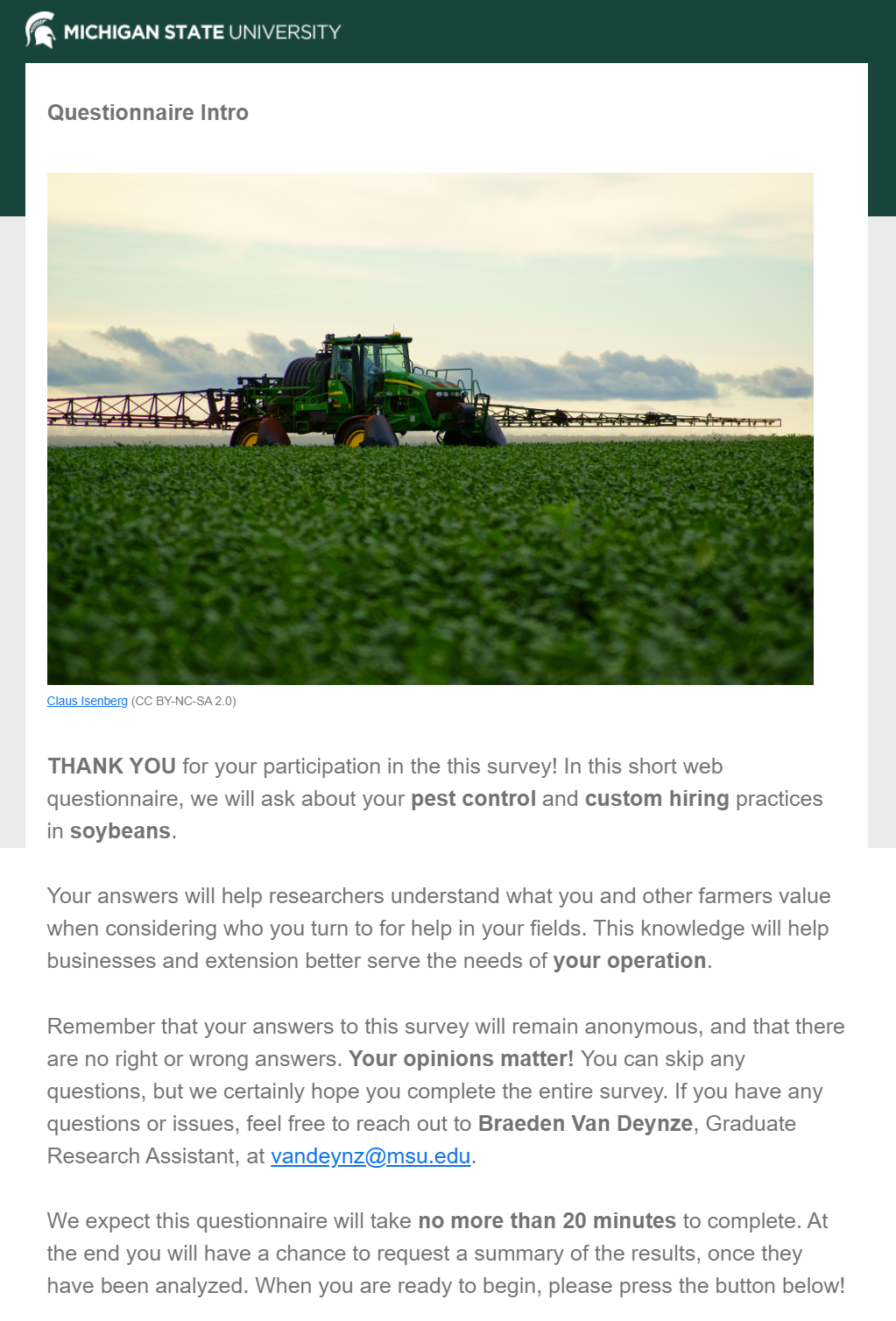


**Figure 3B.1. Screenshots of Online Survey Instrument.**


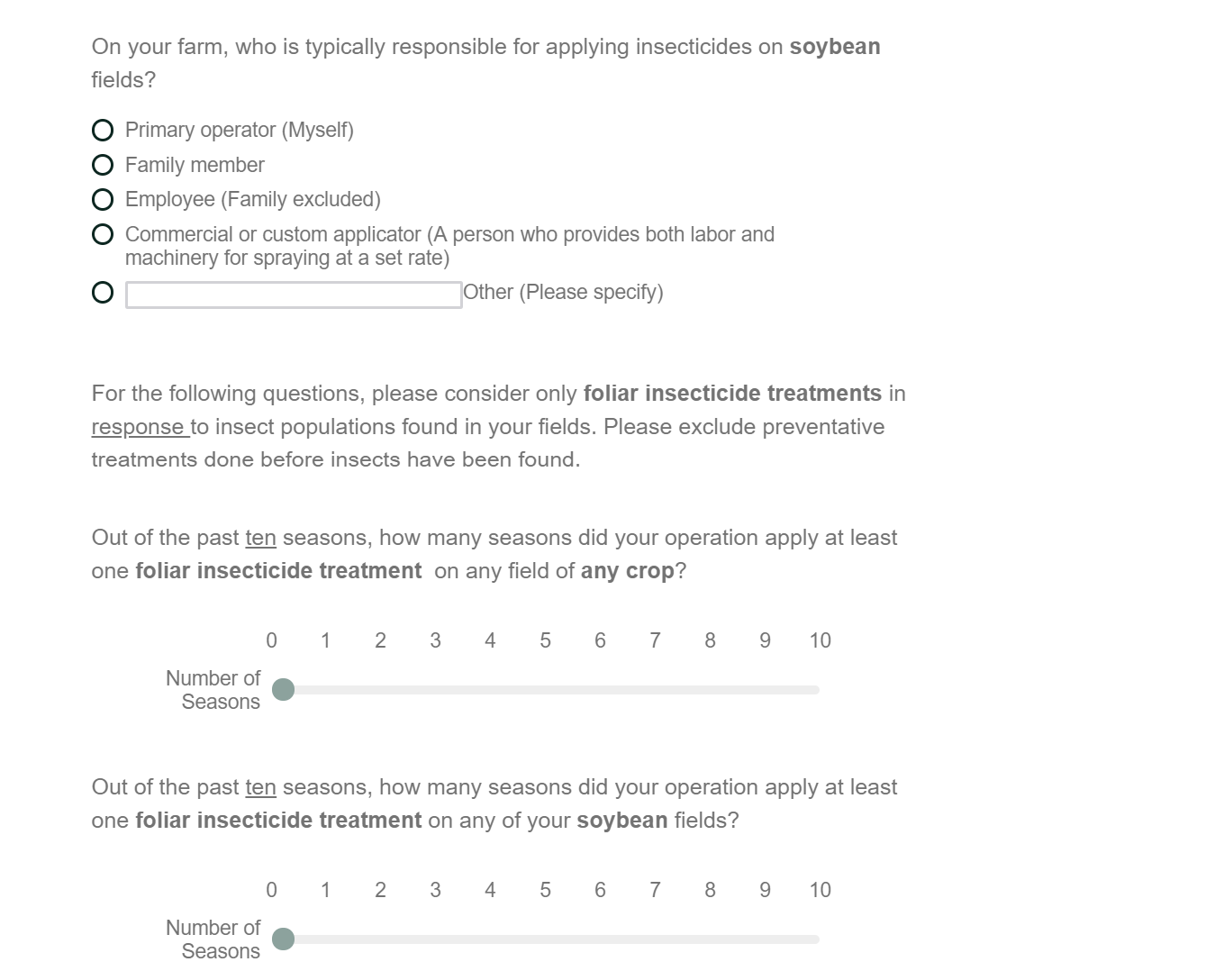


**Figure 3B.1 (cont’d).**


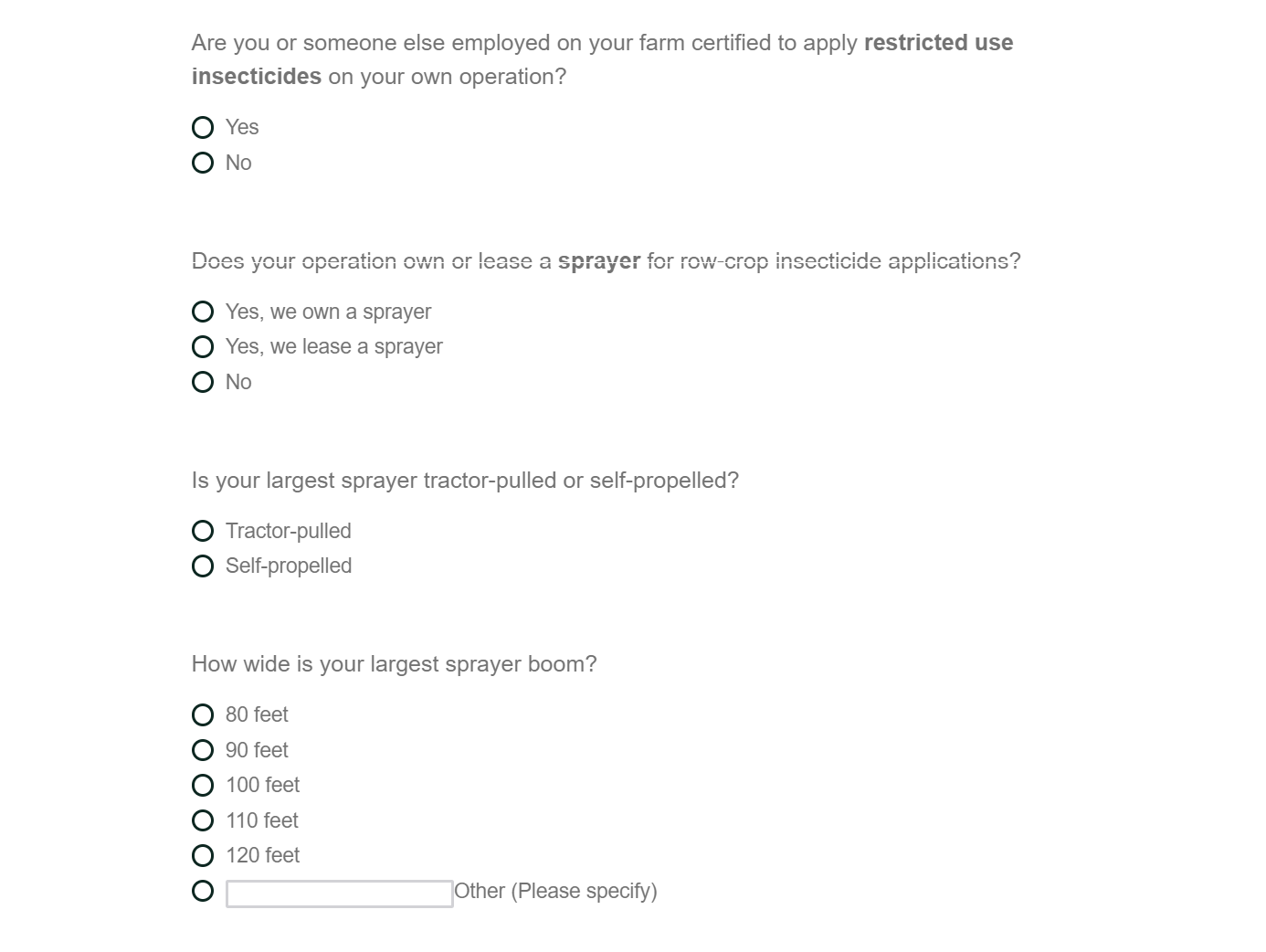


If selected…

**Figure 3B.1 (cont’d).**


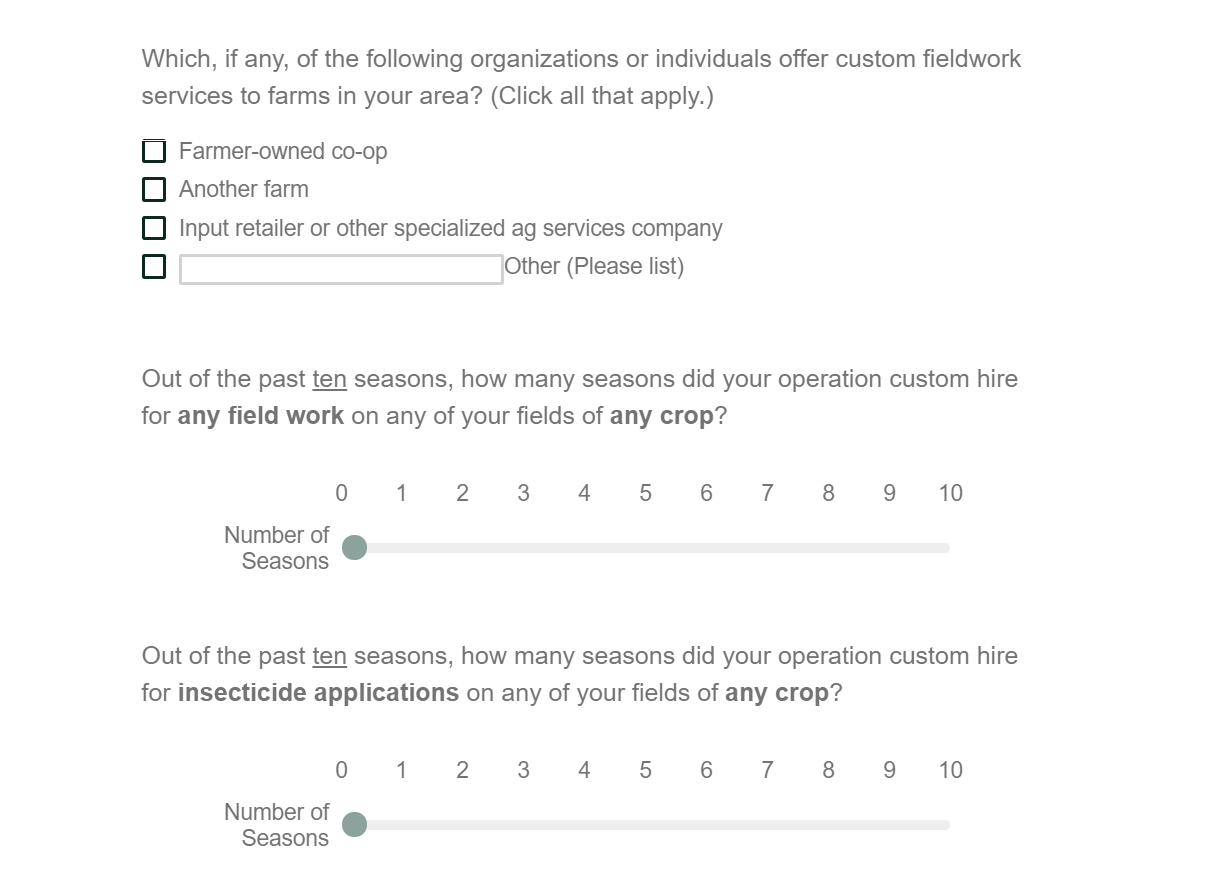


**Figure 3B.1 (cont’d).**


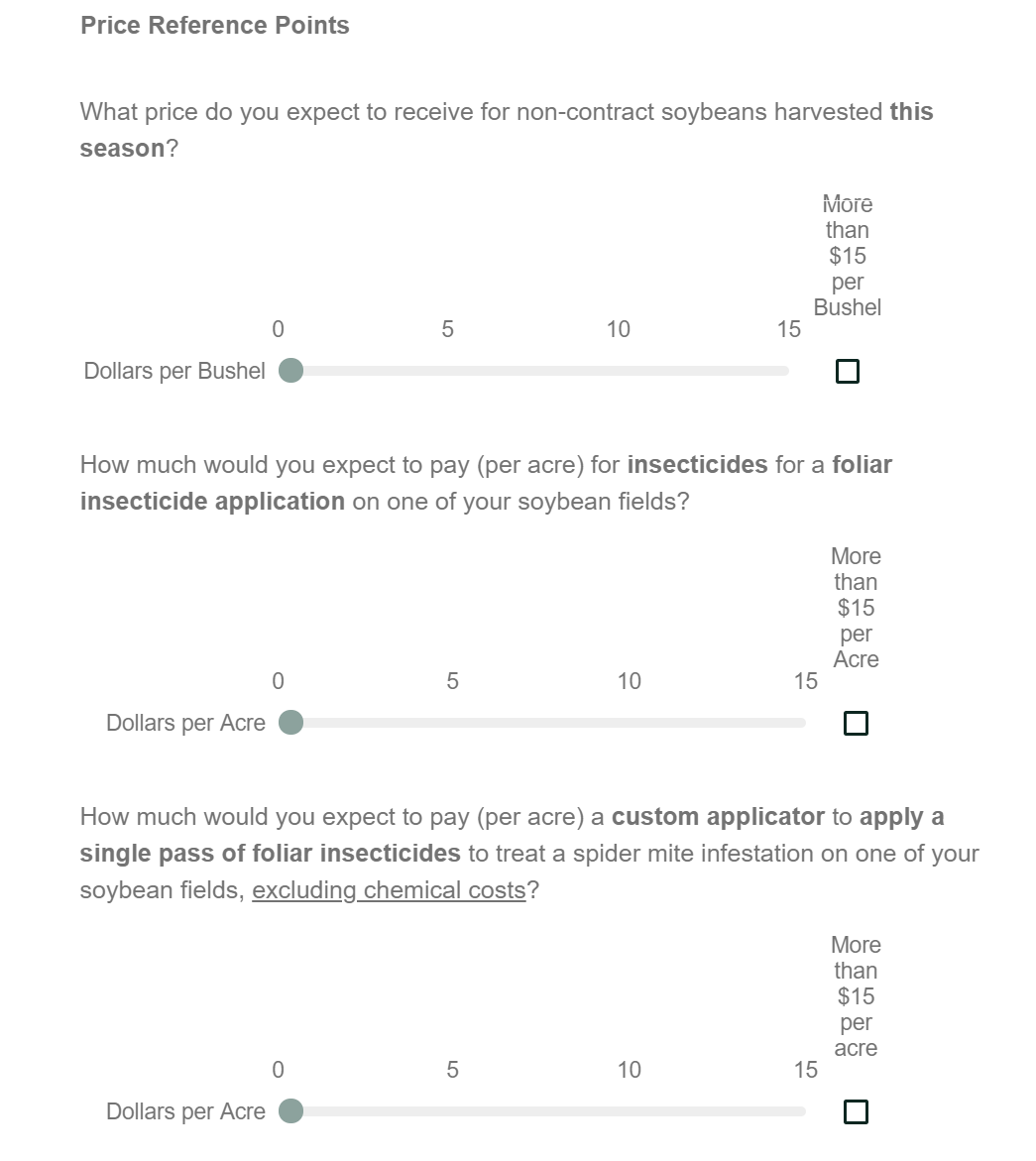


**Figure 3B.1 (cont’d).**


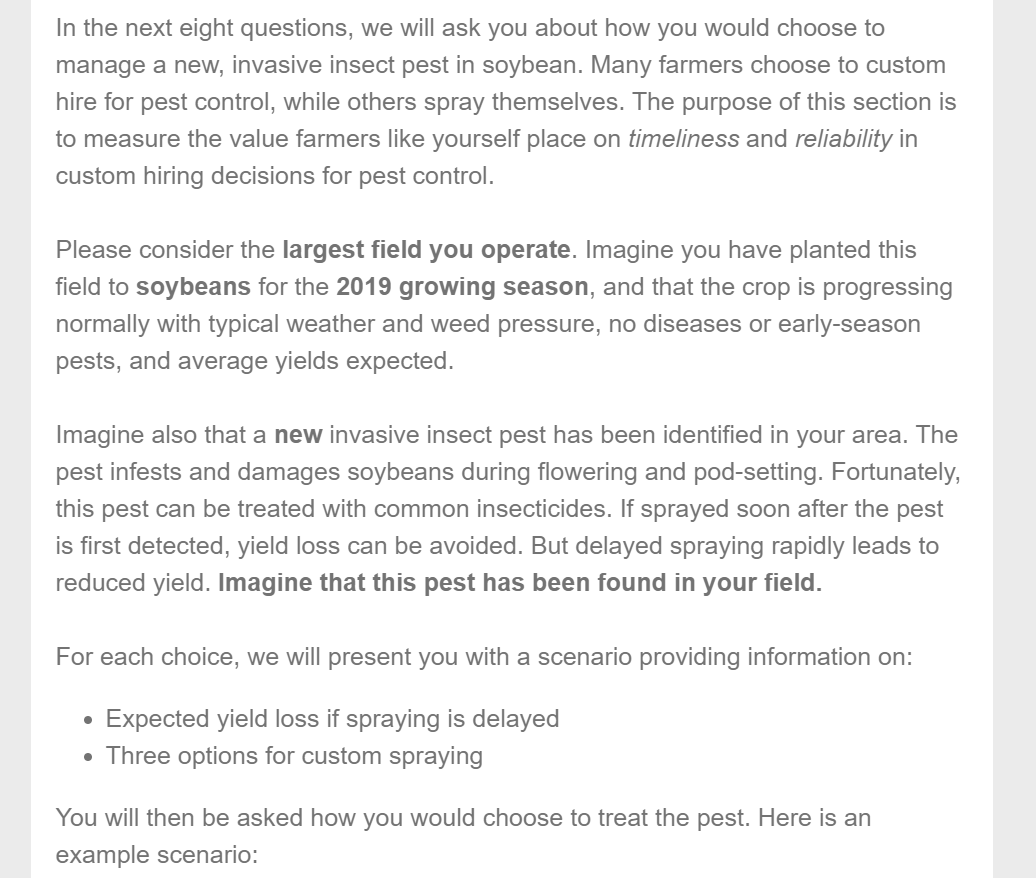


**[PAGE CONTINUES]**

**Figure 3B.1 (cont’d).**


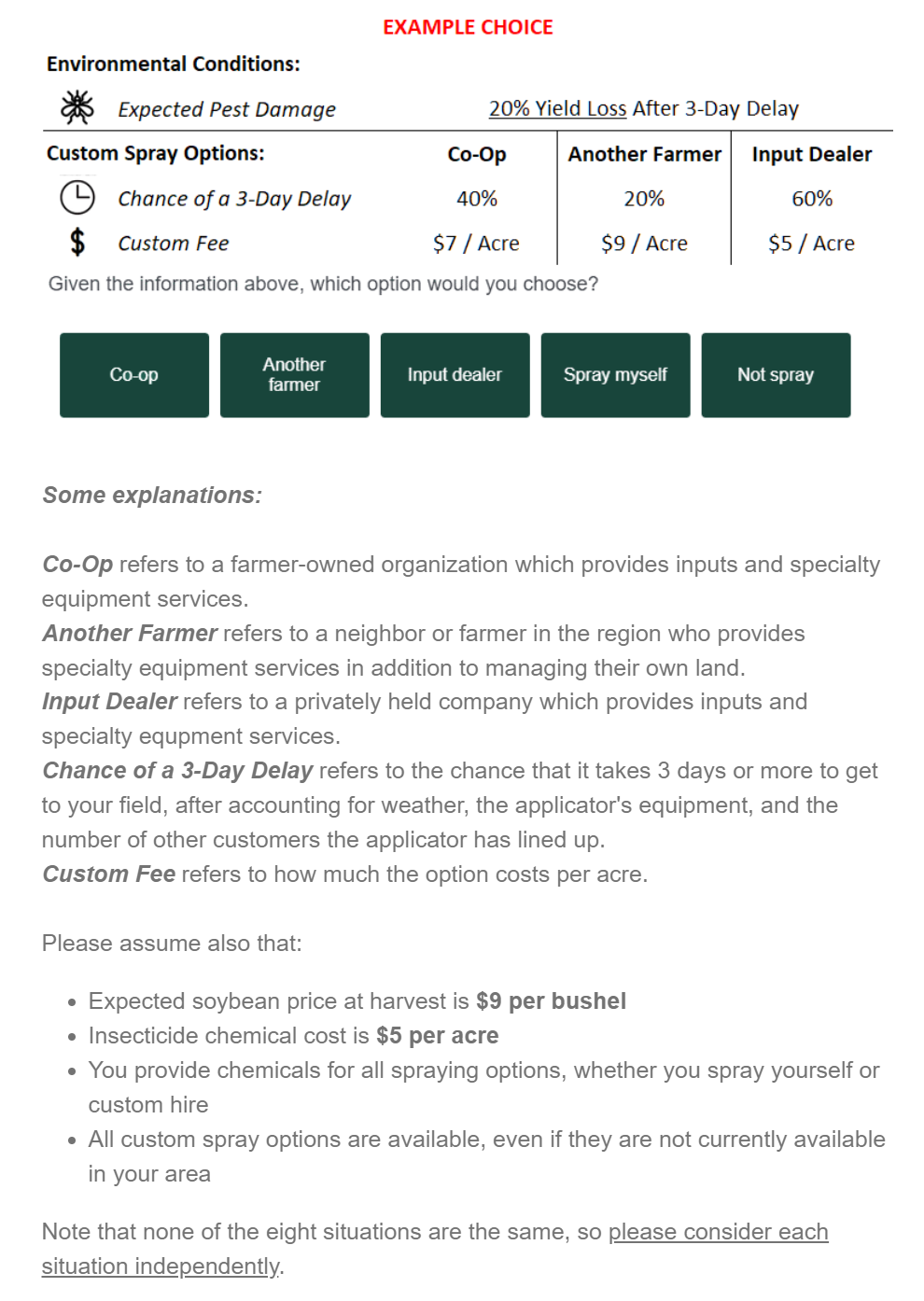


**[CONTINUED FROM PREVIOUS PAGE]**

**Figure 3B.1 (cont’d).**


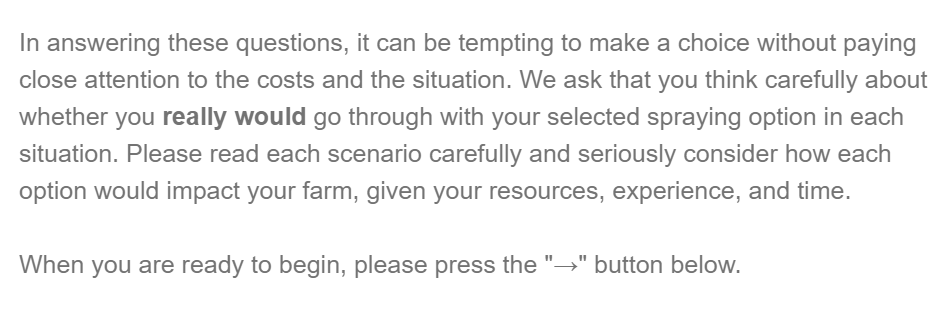


**[FOLLOWED BY 8 CHOICE EXPERIMENT QUESTIONS]**


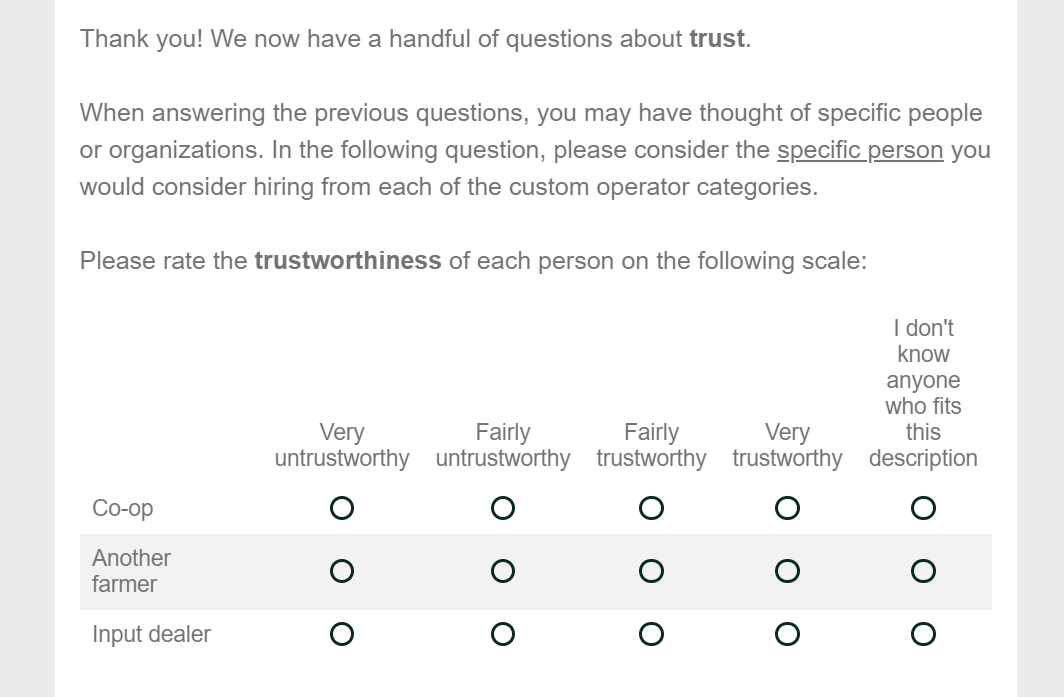


**Figure 3B.1 (cont’d).**


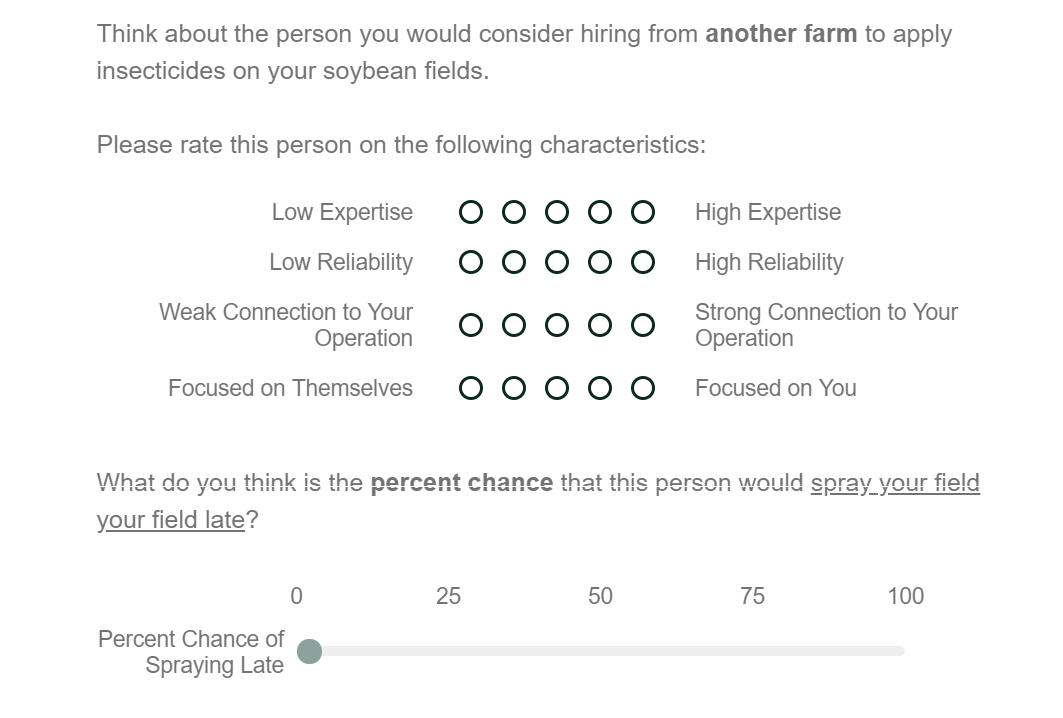


Page repeats with each class of custom operator for which the farmer did not select “I don’t know…” on previous page

**[OPERATOR CLASS]**

**Figure 3B.1 (cont’d).**


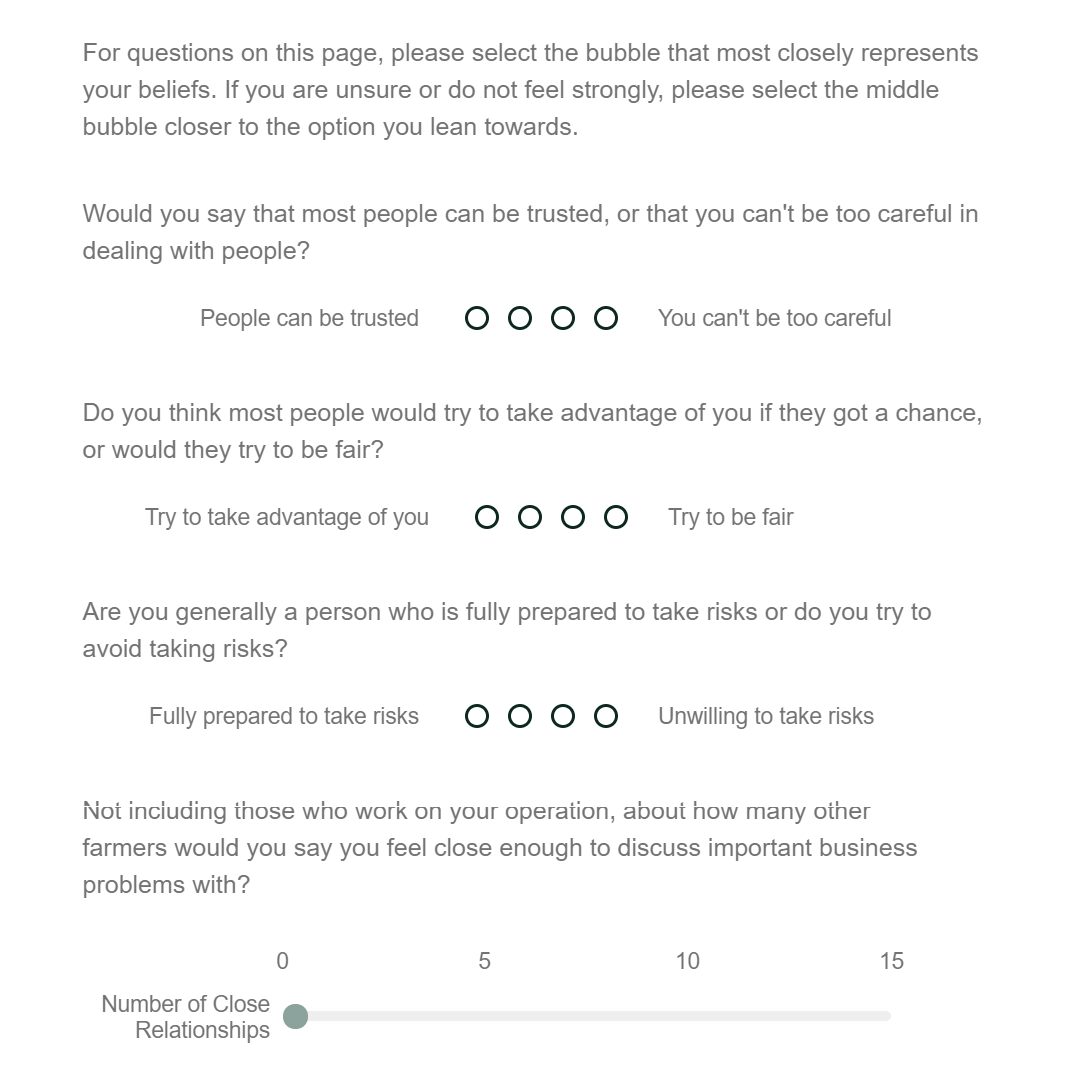


**Figure 3B.1 (cont’d).**


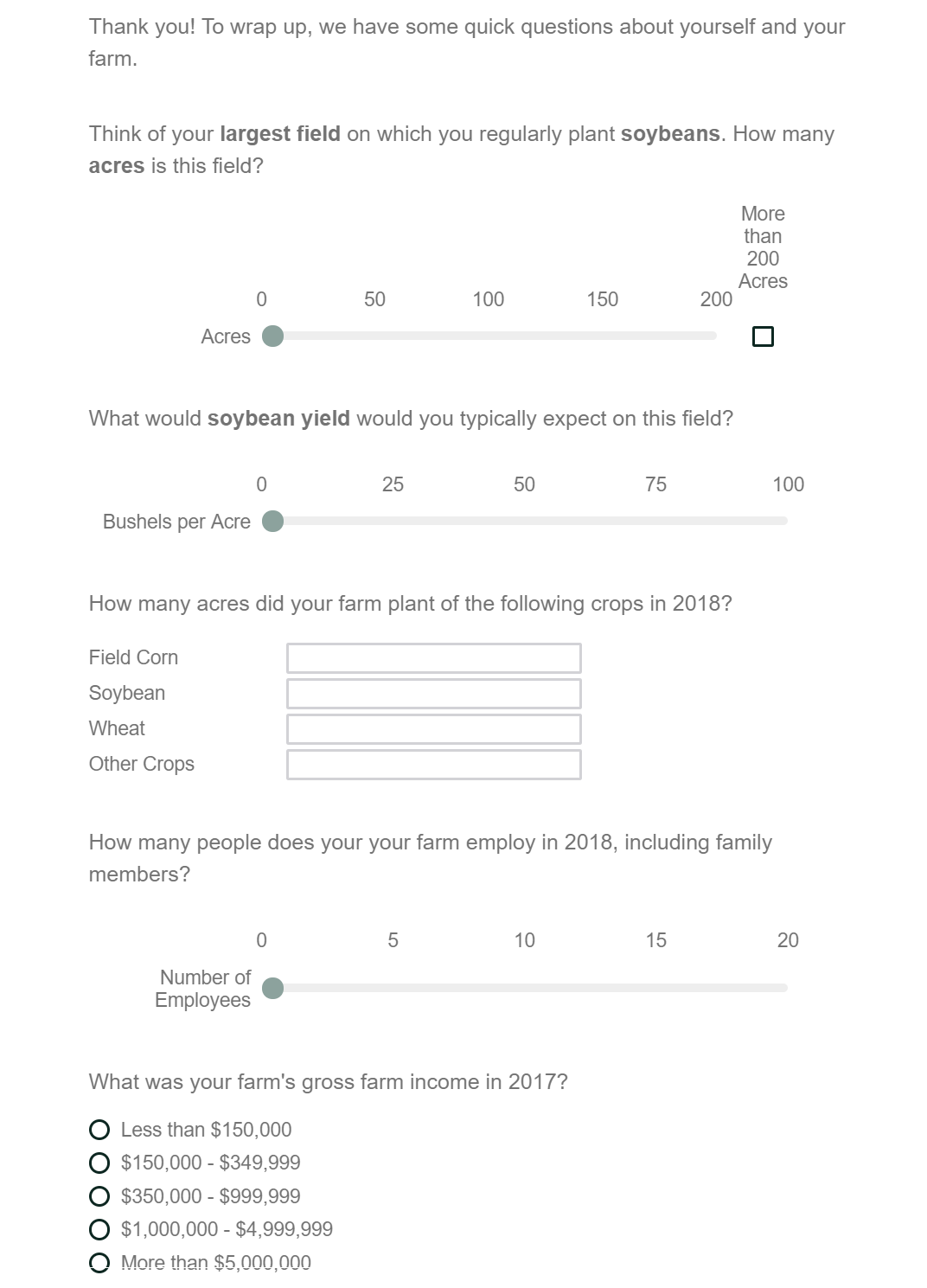


**Figure 3B.1 (cont’d).**


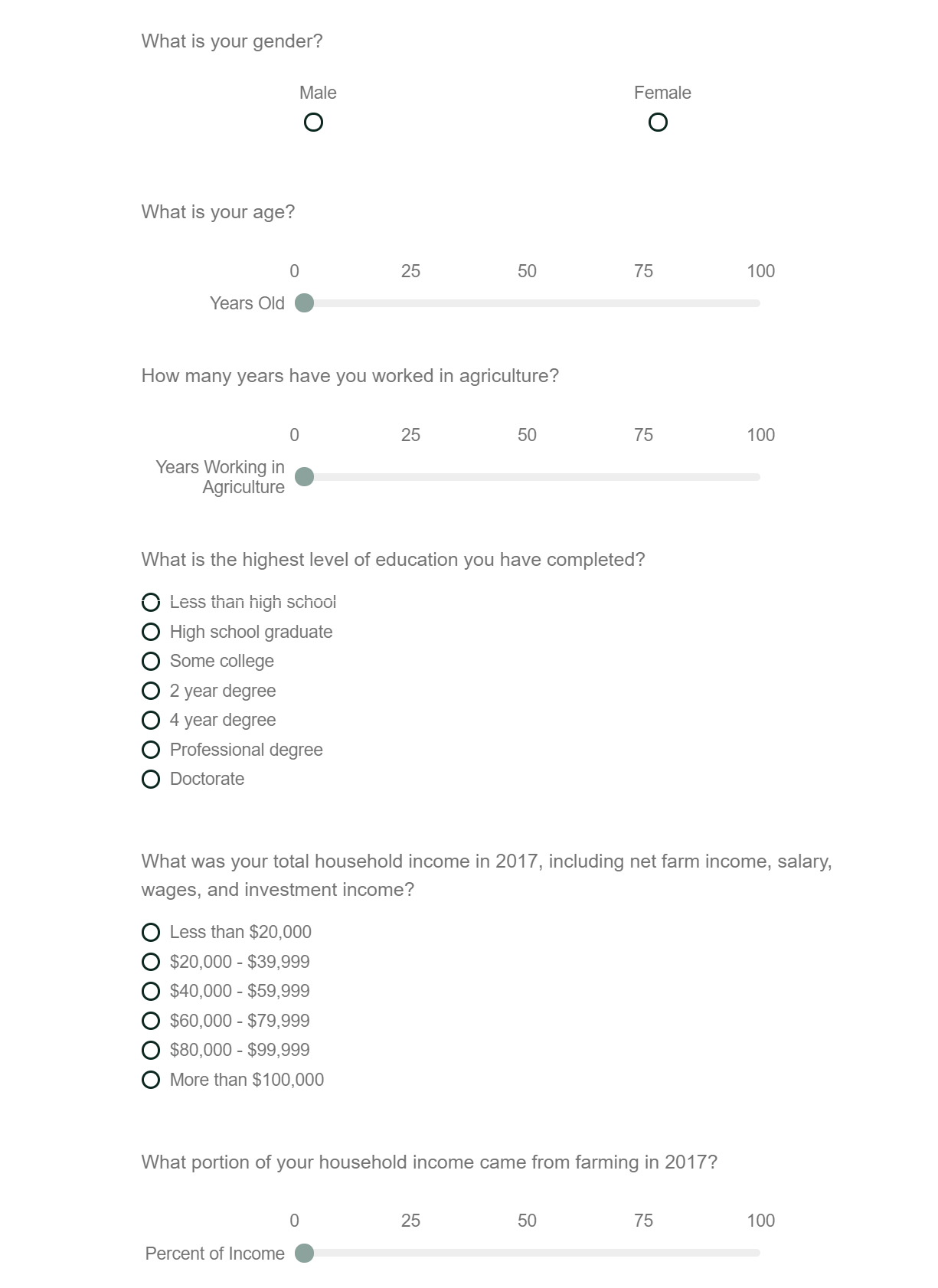


**Figure 3B.1 (cont’d).**

**Appendix B: Survey Deployment and Sample Representativeness**

In this appendix, we assess the representativeness of the sample relative to the target population.

Emails were purchased from the agricultural marketing data company FarmMarketID for 9,290 Illinois farmers, 4,847 Indiana farmers, and 1,895 Michigan farmers, representing all available records with valid emails and 100 or more planted acres of soybeans in 2017. Farmers were emailed three times over the course of a week in August and September of 2018. Emails included a link to an online survey hosted by Qualtrics. To further encourage response, a single letter was mailed to all Michigan farmers and 1,234 of the Indiana farmers directing farmers to visit the online survey.

Of the 16,032 email addresses contacted, 388 were immediately returned as undeliverable due to spam blocking software on the receiving end. These returned emails only represent addresses employing spam blocking software that reports failure of delivery to the sender. We suspect that many more farmers’ email systems blocked all the email contacts without reporting the failure of delivery or filed the email contacts directly to farmers’ spam or junk folders where they were unlikely to be read. This hypothesis is supported by considerably larger response rates in Michigan and Illinois where an additional mail contact was employed.

Choice experiments targeting farmers are frequently limited by small sample sizes (Chèze, David, & Martinet, 2020), including the one presented in this paper. The challenge of obtaining large farmer samples for choice experiment surveys is made more difficult by declining trends in farmer response rates (Johansson, Effland, & Coble, 2017). Further, farmers are often sensitive about discussing pesticide applications due to public concerns over their public environmental and health effects (Chèze et al., 2020).

Direct data on the population of farmers with 100 or more acres of soybeans planted is not publicly available to the best of our knowledge, so we compare demographic characteristics of our sample to results from the 2017 Census of Agriculture over each state, which includes farms smaller than 100 acres and farms that do not grow soybeans (USDA National Agricultural Statistics Service, 2019). Michigan farmers, and to a lesser extent Indiana farmers, are overrepresented in the sample relative to the population of soybean farmers in the three states (51% of the sample versus 23% of total farms of 100 acres or more for Michigan, 34% of the sample versus 28% of total farms of 100 acres or more for Indiana). This is likely the result of issues with email delivery, as Michigan and a subset of Indiana farmers received additional mail invitations to participate in the survey. As a result, the following results should be interpreted as representing mainly the preferences of Michigan and Indiana growers.

The mean expected yield for respondents’ largest soybean fields was 58.1 bushels per acre. This value is considerably higher than the mean yields reported by the Census of Agriculture for Michigan (42 bushels per acre) and Indiana (53 bushels per acre), suggesting that respondents are either more productive than the population or hold optimistic expectations. Our sampling frame was limited to farms of over 100 planted acres of soybeans while the Census of Agriculture reports yields for all growers of soybeans, including the smallest farms which typically do not operate at a commercial scale and therefore often have lower yields. Such smaller farms make up a large portion of the total population targeted by the Census of Agriculture, which may also explain the discrepancy between the mean yield reported by respondents and the mean yield reported by the Census.

Respondents reported planting between 150 and 3,700 total acres across all crops, with a mean of 997 acres planted and median of 697 acres planted. The median planted acres for respondents is considerably higher than the median planted acres for all growers with over 100 acres reported by the Census of Agriculture, which lies in the 220-259 acres range for all three states. The average age of respondents was 58, which is slightly older than the average age reported in the Census of Agriculture for farmers in Michigan and Indiana (56.6 and 55.5 respectively), but roughly equal to the average age of farmers in Illinois (58). Overall, our sample represents larger and more productive farms than those in the population.
